# Proteomics and peptidomics: moving toward precision medicine in urological malignancies

**DOI:** 10.18632/oncotarget.8931

**Published:** 2016-04-22

**Authors:** Ashley Di Meo, Maria D. Pasic, George M. Yousef

**Affiliations:** ^1^ Department of Laboratory Medicine, and The Keenan Research Centre for Biomedical Science at The Li Ka Shing Knowledge Institute, St. Michael's Hospital, Toronto, Canada; ^2^ Department of Laboratory Medicine and Pathobiology, University of Toronto, Toronto, ON, Canada; ^3^ Department of Laboratory Medicine, St. Joseph's Health Centre, Toronto, Ontario, Canada

**Keywords:** prostate cancer, kidney cancer, bladder cancer, personalized medicine, tumor markers

## Abstract

Urological malignancies are a major cause of morbidity and mortality worldwide. Advances in early detection, diagnosis, prognosis and prediction of treatment response can significantly improve patient care. Proteomic and peptidomic profiling studies are at the centre of kidney, prostate and bladder cancer biomarker discovery and have shown great promise for improved clinical assessment. Mass spectrometry (MS) is the most widely employed method for proteomic and peptidomic analyses. A number of MS platforms have been developed to facilitate accurate identification of clinically relevant markers in various complex biological samples including tissue, urine and blood. Furthermore, protein profiling studies have been instrumental in the successful introduction of several diagnostic multimarker tests into the clinic. In this review, we will provide a brief overview of high-throughput technologies for protein and peptide based biomarker discovery. We will also examine the current state of kidney, prostate and bladder cancer biomarker research as well as review the journey toward successful clinical implementation.

## INTRODUCTION

Cancer is a leading cause of death world-wide [1]. Currently, urological tumors are managed based on defined clinicopathological parameters with limited accuracy. Molecular profiling strategies can help guide treatment selection and improve individual patient management through the identification of cancer-specific biomarkers that can subclassify patients into distinct biological subgroups [2, 3]. Different classes of biomarkers have been introduced to the clinic, including proteins, peptides, glycoproteins and hormones [4].

Proteomic and peptidomic profiling methods allow for the identification of cancer-specific proteins and endogenous peptides, respectively. In addition to improving early detection, prognosis and treatment response, proteomic and peptidomic analyses provide an in depth understanding of disease pathology which is key to the discovery of more effective therapies [5–8]. Currently, mass spectrometry (MS) is the most widely employed platform for proteomic and peptidomic analyses. The development of high resolution mass spectrometers has made it feasible to identify hundreds to thousands of potential protein candidates in one experiment. This is critical for promoting translation of clinically relevant markers into the clinic.

This review provides an overview of proteomic and peptidomic strategies for biomarker discovery in addition to outlining the advantages and limitations of each approach. We also provide a summary of proteomic and peptidomic profiling platforms and discuss existing experimental evidence regarding the potential clinical utility of protein and peptide based biomarkers in the diagnosis and prognosis of renal cell carcinoma, prostate cancer and bladder cancer. We also provide an overview of integrative genomics and proteogenomics for cancer biomarker discovery. Finally, we discuss the journey from biomarker discovery to clinical implementation as well as strategies for successful implementation of biomarkers into clinical practice.

## PROTEOMIC AND PEPTIDOMIC ANALYSES

Proteomics refers to the large-scale study of proteins within a biological system. Peptidomics refers to the study of native or endogenous peptides. Unlike proteomics, enzymatic digestion is not required for peptidomic analysis [9]. Various complex biological samples including tissue, nipple aspirate fluid, cerebrospinal fluid, urine, blood, and saliva have been analysed using proteomic strategies [10]. The spectrum of potential utility of proteomic biomarkers is summarized in Table 1. Proteomic analysis has proven to be valuable for the identification of biological markers to improve screening, diagnosis, and prognosis as well as for the identification of novel therapeutic targets [11–13].

**Table 1:** The scope of applications of proteomic cancer biomarkers

Application	Clinical value
Cancer screening	Risk of developing cancer Early detection before the onset of symptoms
Diagnosis	Confirmation of the presence of cancer
Tumor classification and subtyping	Accurate classification of tumors based on biological behaviour
Prognosis	Predict the likely course of a disease (disease aggressiveness)
Prediction of treatment efficiency	Predict treatment response in terms of efficacy and safety, or length of progression-free survival under treatment
Monitoring for recurrence	Predict and detect tumor re-growth after surgical resection or therapeutic intervention
Tumor staging	Indication of tumor development and spread
Tumor localization and directing chemo- or radio-therapeutic agents	Predict optimal therapeutic intervention
Monitoring the response of therapy	Indication of response to therapy

## ADVANTAGES AND LIMITATIONS

The advantages and limitations of proteomics and peptidomics are summarized in Table 2. Proteomics enables a more in depth understanding of disease pathology compared to traditional genomic or tanscriptomic studies through the ability to analyze dynamic protein expression, post-translational modifications (PTMs), cellular and sub-cellular localization and protein-protein interactions. Proteomics also allows for the detection of disease-specific protein isoforms [5, 6]. Furthermore, high-throughput proteomics data can be utilized for functional analysis, including protein ontology, protein-protein interactions, protein-DNA interactions and pathway analysis. Protein sequence alignments can also provide information regarding sequence similarity in addition to homology [14].

**Table 2:** Advantages and limitations of proteomic and peptidomic analyses

**Proteomics**	**Advantages** Allows an in-depth analysis of dynamic protein expression, PTMs, cellular and sub-cellular protein distribution, and protein-protein interaction Allows the detection of protein isoforms Accurately reflects actual cellular processes Can be utilized for functional analysis Allows the discovery of protein sequence similarity
	**Limitations** Lack of validation studies Technologies (mass spectrometry) require specialized staff Susceptible to biological variability Reduced detection of low abundant proteins due to the presence of high abundant proteins (masking effect) Digested protein fragments may match to several proteins Digestion-induced modifications may result in a failure to identify specific interactions Requires sophisticated bioinformatic algorithms for accurate analysis
**Peptidomics**	**Advantages** Provides insight regarding proteolytic activity Allows for the study of the disease microenvironment Requires no chemical or enzymatic digestion for sample processing
	**Limitations** Lack of validation studies Technologies (mass spectrometry) require specialized staff Susceptible to biological variability Low molecular weight (LMW) peptides are low abundant LMW peptides may associate with highly abundant proteins, reducing their detection Data analysis is challenging due to the absence of chemical or enzymatic digestion Endogenous peptides may match to several proteins Requires sophisticated bioinformatic algorithms for accurate analysis

Failure to validate initial discoveries and a lack of a universal method for sample handling are the biggest limitations of proteomic analysis. The correct use of statistics is also important, especially when handling high-throughput proteomics data where the chance of obtaining false positive results can be significantly higher. Another limitation of proteomic studies is that significant biomarkers might reflect a non-specific systemic response. For instance, candidate biomarkers may be part of a generalized biological response (stress, inflammatory, etc.), which would have little diagnostic value due to a lack of specificity [15]. To address this issue, prospective studies should be used as a gold standard for validation. In addition, the presence of highly abundant proteins can obscure the detection of the low abundant ones. Sample preparation techniques that enrich for low abundant proteins can help overcome this limitation. Biological variability between samples is another challenge facing proteomic studies as this can complicate protein profiling strategies. To minimize bias associated with biological variability and to ensure biomarker specificity it is important to select appropriate study subjects. This can be achieved by taking into consideration age, gender, subject status (body mass index, hypertension, and smoking) as well as the presence of other comorbidities [16]. In the case of urine, variability in fluid consumption can be normalized by adjusting creatinine levels [5].

Peptidomic studies can provide additional information regarding the proteolytic activities occurring in a pathophysiological context and therefore a deeper understanding of the molecular mechanisms of disease. In addition, the low molecular weight (LMW) peptidome likely contains clinically relevant biomarkers, as LMW peptides are able to passively diffuse across endothelial barriers. This is advantageous as it provides an opportunity to study the disease microenvironment which can offer more information regarding early pathophysiological changes. Another advantage of peptidomics is that no chemical or enzymatic digestion is required for analysis [17].

Although peptidomic studies offer a promising strategy for the discovery of novel cancer biomarkers, they have a number of limitations. LMW proteins are present at relatively low abundance. Also, there is potential for LMW peptides to associate with highly abundant proteins which would inevitably result in a loss of information [18]. Furthermore, a loss of information can occur due to the tendency of highly abundant proteins to suppress the signal of low abundant peptides [19]. This masking effect can be overcome through the enrichment for LMW peptides during sample preparation. Identification of endogenous peptides during analysis is also more complex as the site of enzymatic digestion cannot be specified. Thus, the criteria for peptide identification have to be more stringent than those for proteomic identification [20].

## PROTEIN PROFILING APPROACHES

Protein profiling requires the use of highly specific and sensitive detection methods for accurate analysis. This is because the identification of clinically relevant biomarkers involves the analysis of complex biological samples such as urine, blood, saliva, and cerebrospinal fluid, which often display a wide dynamic range of protein concentrations. Protein profiling approaches can be classified into discovery-based or target-based. In discovery-based strategies, the investigator has no previous knowledge of a potential candidate, whereas in target-based strategies the researcher has already identified potential candidates [21].

## MASS SPECTROMETRY

**Figure 1:**
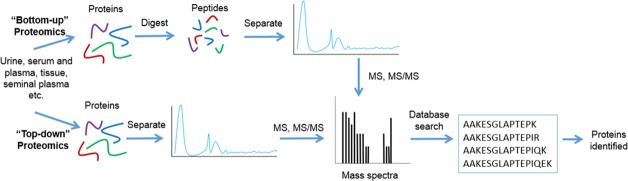
Workflow for bottom-up and top-down proteomics For bottom-up proteomics proteins are separated and are either chemically or enzymatically digested to generate peptides. These peptides are then analyzed using mass spectrometry. The mass spectra of an individual peptide is then matched to a sequence through a protein database search. In the case of top-down proteomics, proteins are separated and directly, without chemical or enzymatic digestion, analyzed using mass spectrometry. Again, the mass spectra generated is then matched to a sequence through a protein database search. Both bottom-up and top-down proteomic approaches result in protein identification.

MS has been widely employed for the study of proteins and low molecular weight peptides. MS-based protein profiling strategies can follow a bottom-up or top-down approach, as shown in Figure 1. Traditionally, MS-based studies follow a bottom-up approach, which involves the chemical or enzymatic digestion of proteins prior to their introduction to the mass spectrometer [22]. Conversely, top-down proteomics involves the characterization of intact proteins. This strategy allows for better characterization of PTMs and protein isoforms [22].

**Figure 2:**
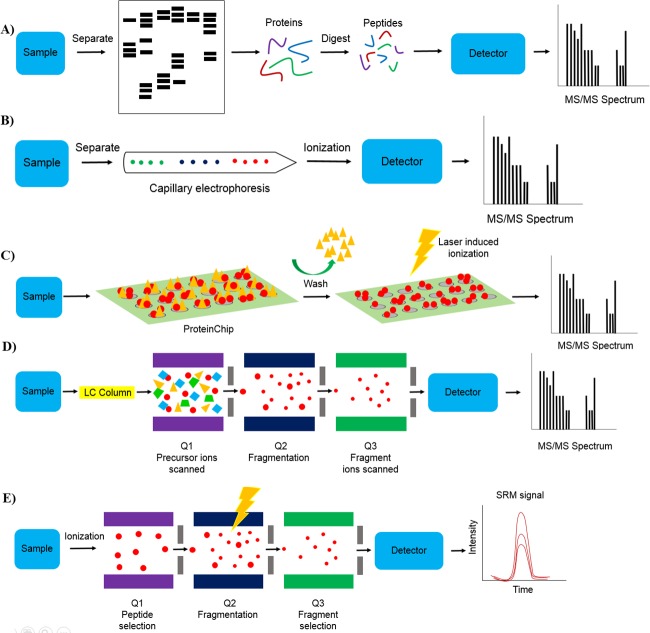
Mass spectrometry technologies **A.** Two dimensional gel electrophoresis mass spectrometry (2-DE-MS). Sample is separated using gel electrophoresis followed by in gel digestion or out-of-gel digestion of proteins and mass spectrometry analysis, **B.** capillary electrophoresis mass spectrometry (CE-MS). Sample is separated using capillary electrophoresis followed by mass spectrometry analysis, **C.** Surface-enhanced laser desorption/ionization / matrix-assisted laser desorption/ionization mass spectrometry (SELDI- and MALDI-MS). Sample is applied to a ProteinChip and washed to remove non-specifically bound substrates followed by mass spectrometry analysis, **D.** Liquid chromatography coupled to tandem mass spectrometry (LC-MS/MS). Sample is separated using liquid chromatography followed by ionization and mass spectrometry analysis. For tandem mass spectrometry, precursor ions with a known mass are scanned in Q1 (first mass filter) followed by precursor ion fragmentation in Q2 and fragment ion scanning in Q3 (second mass filter), and **E.** selected reaction monitoring mass spectrometry (SRM-MS). For targeted protein quantification, target ions are selected in Q1 followed by target ion fragmentation in Q2 and fragment ion selection in Q3.

There are a number of different MS technologies that allow for the identification of clinically relevant biomarkers, as shown in Figure 2. Furthermore, the advantages and limitations of these different technologies are summarized in Table 3. Two-dimensional gel electrophoresis mass spectrometry (2DE-MS) used to be a traditional protein profiling strategy [23]. To correct for poor throughput of 2DE-MS, two-dimensional difference gel electrophoresis mass spectrometry (2D-DIGE-MS) was developed [24]. Capillary electrophoresis-mass spectrometry (CE-MS) is another protein profiling technique. It is a low cost technique that has successfully been employed in the clinic for peptide identification in biological samples for various diseases [25]. Other MS-based methods include the use of protein chip technology coupled to surface-enhanced laser desorption/ionization mass spectrometry (SELDI-MS) as well as matrix-assisted laser desorption/ionization mass spectrometry (MALDI-MS). Both techniques emerged as tools for protein profiling with tremendous potential for clinical diagnosis [26]. SELDI-MS has been successfully used to identify highly sensitive and specific potential biomarkers for the diagnosis of urological cancers [27]. MALDI- imaging, a relatively new application of MALDI-MS has also been applied to the identification of clinically relevant biomarkers for a number of tumors [28]. Liquid chromatography coupled to mass spectrometry (LC-MS) is another platform that allows for the detection of proteins with greater depth, dynamic range, and enhanced accuracy. Liquid chromatography coupled to tandem mass spectrometry (LC-MS/MS) is an extension of the LC-MS platform. LC-MS/MS is a high-throughput platform with high reproducibility, dynamic range and accuracy [29]. In addition, selected reaction monitoring (SRM)-MS has been developed as a promising strategy for candidate biomarker validation [30] SRM-MS is a targeted MS approach that displays superior sensitivity, specificity and reproducibility. Assay development is quick compared to immunoassays and protocols can be easily transferred among laboratories [31]. It is also able to detect and quantify specific PTMs which cannot be achieved using antibody-based assays [32]. This highlights the potential clinical application of SRM-MS as specific PTMs may have diagnostic value [33].

**Table 3:** Mass spectrometry techniques

Name	Abbreviation	Advantages	Limitations
**Discovery**			
Two-dimensional gel electrophoresis mass spectrometry	2-DE-MS	Suitable for analysis of large molecules	Labor intensive Requires large sample volume Low throughput
Two-dimensional difference gel electrophoresis mass spectrometry	2-DIGE-MS	Minimal gel-to-gel variation Improved sensitivity Facilitates spot matching	Unable to resolve highly basic, acidic, or hydrophobic proteins
Capillary electrophoresis mass spectrometry	CE-MS	Fast separation High resolution and reproducibility Low cost Successfully employed in the clinic	Long processing times Limited loading capacity
Surface-enhanced laser desorption/ionization time of flight mass spectrometry	SELDI-TOF-MS	Requires low sample volume High sensitivity Covers a wide mass range	Immobilization results in a loss of information Low resolution
Matrix-assisted laser desorption/ionization time of flight mass spectrometry	MALDI-TOF-MS	Requires low sample volume High sensitivity Inexpensive Covers a wide mass range	Low resolution Poor fragmentation Immobilization results in a loss of information
Liquid chromatography mass spectrometry	LC-MS	High depth and dynamic range Enhanced accuracy	Restricted mass range Sensitive to interfering compounds
Liquid chromatography coupled to tandem mass spectrometry	LC-MS/MS	High reproducibility and dynamic range Improved accuracy High loading capacity	High cost Requires high level of expertise
**Validation**			
Selected reaction monitoring	SRM	Superior multiplexing capabilities High sensitivity and specificity High reproducibility Short time for assay development Easily transferred	Lack sufficient sensitivity for quantification of low abundance proteins or protein modifications

## ALTERNATIVE PROTEIN PROFILING APPROACHES

Other protein profiling approaches include Western blot, ELISA, immunohistochemistry, tissue microarrays, and protein microarrays. However, these methods are not suitable for large-scale analysis due to poor throughput and problems associated with the availability of commercial antibodies [15, 34]. Protein microarrays allow for simultaneous profiling of hundreds or thousands of known proteins [34]. There are three types of protein arrays that are available; analytical, functional and reverse phase microarrays. Analytical microarrays are used to profile complex mixtures of proteins and are often used to measure binding affinities, specificities, and expression levels of proteins [35]. Functional arrays are used to study biochemical activities including protein-protein interactions, protein-DNA interactions, posttranslational modifications and enzyme-substrate relationships [36]. Reverse-phase arrays are a relatively new, sensitive, and high-throughput technology [37]. In this technology, a cell lysate is immobilized and probed with antibodies specific to target proteins [37].

## CLINICAL APPLICATION IN CANCER

### Renal cell carcinoma

Renal cell carcinoma (RCC) is the most common neoplasm of the adult kidney. The disease is histopathologically heterogeneous, comprising of a number of subtypes including clear cell RCC (ccRCC) (70-75% of cases), papillary RCC (pRCC) (10-16%), and chromophobe RCC (chRCC) (5%), in addition to others [38]. Independent groups have demonstrated that protein and peptide profiling strategies offer promise for improved diagnosis and prognosis [39]. An overview of proteomic and peptidomic studies is summarized in Table 4. The studies listed demonstrate a great advantage of the newer technologies in identifying a much larger number of proteins compared to previous generation techniques.

**Table 4:** Identified proteins and peptides in renal cell carcinoma, prostate cancer, and bladder cancer

Proteins/peptides	Cancer/control (size)	Sample	Clinical application	Ref
**RENAL CELL CARCINOMA**				
**Proteomic studies**				
PFN1, 14-3-3 ζ/δ, and GAL1	Metastatic RCC (6)/primary RCC (6)	Tissue	Prognostic	[13]
ENO1, LDHA, HSPB1/ Hsp27, HSPE1	ccRCC (199)/normal (30)	Tissue	Diagnostic	[42]
ADRP, CORO1A	Primary RCC (8)/normal (8)	Tissue	Diagnostic	[43]
FABP7, HBA1, HBB, etc.	Progressive ccRCC (10)/non-progressive ccRCC (10)	Tissue	Prognostic	[50]
RCN1	RCC (7)/normal (7)	Tissue	Diagnostic	[88]
**Peptidomic studies**				
CO1A2, B2MG, CO1A1, ATNG, etc.	RCC (40)/control (68)	Urine	Diagnostic	[16]
SDPR, ZYX, SRGN, and TMSL3	ccRCC (85)/benign lesions (12)/control (92)	Serum	Diagnostic	[45]
CUBN	RCC (30)/control (30)	Serum	Diagnostic	[46]
**PROSTATE CANCER**				
**Proteomic studies**				
MIC1	PCa/benign prostate hyperplasia*	Tissue	Diagnostic	[11]
β-MSMB	PCa (25)/ benign (27)	Urine	Diagnostic	[58]
BLVRB	PCa (13)/ benign prostatic hyperplasia (2)/normal (15)	Tissue	Diagnostic	[61]
PDCD6IP, FASN, XPO1, and ENO1	PCa cell line/prostate epithelial cell line	Cell line	Diagnostic	[62]
TM256, LAMTOR, VAT1, and ADIRF	PCa (16)/control (15)	Urine	Diagnostic	[63]
**Peptidomic studies**				
FXYD2, CO1A3, CO1A1, and SPR1	PCa (51)/normal (35)	Urine	Diagnostic	[59]
LAMA1	Gleason score 6 (23)/Gleason score 8 to 9(23)	Tissue	Prognostic	[64]
B3GNT1, ACPP, STAB2, GIMAP6, etc	PCa (70)/prostatic hyperplasia (21)/chronic prostatitis (25)/control (9)	Seminal plasma	Diagnostic	[69]
**BLADDER CANCER**				
**Proteomic studies**				
APOA1, APOA2, SERPIND1 etc	BCa (23)/control (14)	Urine	Diagnostic	[12]
SAA4 and Pro-EGF	BCa (12)/control (12)	Urine	Diagnostic	[70]
AFM, ADIPOQ, APOA2, CP, etc.	BCa (76)/control (57)/urinary tract infection or hematuria (23)	Urine	Diagnostic	[71]
**Peptidomic studies**				
S100A8 and S100A4	High-grade (32)/low-grade (33)	Serum	Prognostic	[77]
ALB, FGA, FGB, HBA, TTR	Muscle-invasive (162)/non-invasive (589)	Urine	Prognostic	[79]
PGRMC2, CO1A1, UMOD, CO1A3	Muscle invasive BCa (56)/non-invasive BCa (71)	Urine	Prognostic	[80]
Fibrinopeptide A	Urothelial carcinoma (46)/normal (33)	Urine	Diagnostic	[89]

* Sample size is not indicated. Study was done using a whole-mount FFPE prostate tissue block taken from a radical prostatectomy. The tissue displayed a range of well, moderate, and poorly differentiated carcinoma of intermediate grade, prostatic intraepithelial neoplasia, and glandular hyperplasia.

Early-stage RCC is often incidentally detected as patients rarely present with signs, symptoms, or laboratory abnormalities [38, 40]. In addition, about 20% of incidentally detected lesions are benign [41]. The discovery of diagnostic markers is essential to guide patient management. A study aimed at identifying diagnostic markers for RCC assessed variations in protein levels in fresh frozen tissue from patients with ccRCC and matched controls. Using iTRAQ labeling and LC-MS/MS, this group identified ENO1, LDHA, HSPB1/Hsp27, and HSPE1 [42]. Alternatively, PLIN2 and CORO1A were found to be significantly elevated in primary RCC specimens compared to adjacent normal fresh frozen tissues using a label-free LC-MS/MS approach [43]. By applying a label-free quantitative proteomics approach, Zhao et al. identified mitochondrial proteins, ACAT1 and MnSOD as potential tissue-based markers for ccRCC diagnosis [33]. Interestingly, the authors observed decreased ACAT1 expression in ccRCC compared with adjacent normal tissue which was consistent with a previous report by White et al. [33, 42]. Using SELDI-TOF-MS, Neurrula et al. established a non-invasive serum diagnostic model. Five serum proteins were identified to have a high predictive value for ccRCC with 88.8% sensitivity and 91.0% specificity [44]. Frantzi et al. employed CE-MS to assess alterations in the urinary peptidomic signature in patients with RCC and controls. The authors identified a classifier of 40 endogenous peptides with 80% sensitivity and 87% specificity [16]. Using MALDI-TOF and LC-MS/MS, one group identified a highly discriminating peptide cluster in serum able to differentiate malignant from benign renal masses and healthy controls [45]. Another study identified a diagnostic peptide signature that could differentiate patients with ccRCC from healthy controls by LC-MS/MS [46]. Chinello et al. identified a cluster of 12 peptide signals by MALDI-TOF-MS in urine that could differentiate malignant tumors from benign lesions and controls with 76% sensitivity and 87% specificity. In addition, this group identified a second peptide cluster that could distinguish ccRCC from controls with 84% sensitivity and 91% specificity [47]. Although there is overlap among independent groups, differences in significantly altered markers has also been observed. The use of different RCC subtypes and the lack of standardized methods for sample handling among studies can influence this. Standardized methods for sample handling are necessary for proteomics to be effectively introduced into the clinic. This will guarantee reproducibility of results across various institutions and ensure that observed changes are attributable to disease states and not workflow variability, as highlighted in previous publications [48]. It is also critical to consider early stage cancer where biomarker levels are expected to be much lower. Moreover, independent validation is essential to determine whether there is sufficient evidence to support the clinical utility of a given candidate biomarker. This is more important when we take into consideration that the majority of discovery based studies use a small set of samples which can lead to model overfitting. To ensure success of biomarker studies and to improve the odds of finding a clinically relevant marker, the maximal number of candidate biomarkers should be verified.

Prognosis of RCC is quite variable. Currently, prognosis is based on a multivariate analysis developed at Memorial Sloan Kettering [49], which has limited accuracy for predicting aggressive behaviour. The ability to accurately predict progression would significantly impact patient care. A study aimed at identifying prognostic markers for RCC examined alterations in protein levels in fresh frozen tissue from patients with primary ccRCC and metastatic ccRCC. Using iTRAQ labeling and LC-MS/MS, this group identified PFN1, 14-3-3 ζ/Δ, and GAL1 as potential prognostic markers [13]. Another study assessed ccRCC progression in fresh frozen tissue from patients who displayed disease recurrence and metastasis within 3 years of surgery and patients who remained progression free for more than 5 years following surgery. Using MALD-MS imaging and LC-MS/MS, the authors identified a panel of 26 proteins that could distinguish non-tumors and recurrent ccRCC progressors from non-progresssors [50]. By applying a non-invasive peptidomic approach, Chinello et al. identified peptides whose urinary abundance varied according to tumor size, stage and grade. Identified peptides corresponded to proteins previously reported to play a role in RCC progression including NOTCH2, ADAM19, SAFB2 and AGP [51]. To identify markers predictive for poor prognosis, Sandim et al. assessed the urinary proteome of ccRCC patients using three independent MS platforms. The authors identified decreased expression of KNG1, UMOD, APOD, polyubiquitin (UBB and UBC) and CD59 in patients with poor prognosis compared to those with good prognosis [52]. Standardization of technology platforms and sample preparation will have to be achieved before the clinical utility of these markers can be realized. Moreover, it seems a biomarker panel is likely to replace individual markers which show limited sensitivity and specificity.

### Prostate cancer

Prostate cancer (PCa) is the second leading cause of cancer deaths in men. Prostate specific antigen (PSA) is the most commonly used biomarker for PCa. However, the moderate specificity of PSA has raised concerns regarding unnecessary biopsies as well as over-diagnosis and over-treatment of PCa [53]. Several clinical multimarker tests are currently available for PCa. The Prostate Health Index (PHI) is a blood test combining total PSA, free PSA, and [2] proPSA for PCa detection. PHI was found to outperform its individual components for PCa detection as well as predict likelihood of progression during active surveillance [54]. Metamark genetics identified an initial set of 12 biomarkers that was further developed into an 8 marker proteomic assay for formalin fixed paraffin embedded tissue biopsies. This proteomic signature was found to be an independent predictor of “favorable” versus “non-favorable” pathology of prostate cancer patients. It can also improve the precision of clinical decision making following biopsy [55, 56]. Although proteomics from formalin fixed tissues is promising, it still awaits independent validation of the efficiency and reproducibility of the technology. Comparison between fresh and formalin fixed tissues needs to be done. The 4KScore is another multimarker blood test that combines measurement of four kallikreins including total PSA, free PSA, intact PSA, and human kallikrein 2 for assessment of significant (Gleason > 7) PCa before biopsy [57]. Moreover, protein profiling strategies continue to be adopted by independent groups for PCa diagnosis and prognosis. An overview of proteomic and peptidomic studies that have been applied to PCa is summarized in Table 4.

Prostate specific antigen (PSA) is the most commonly used marker for PCa diagnosis. However, the moderate specificity of PSA has raised concerns regarding over-diagnosis and overtreatment of PCa. Several independent protein profiling studies report improved sensitivity of detection when protein markers are combined with PSA levels. Flatley et al. identified β-MSMB using MALDI-MS profiling of pre- and post-digital rectal examination urines from patients with PCa and various benign prostatic conditions. The authors found that when β-MSMB levels were combined with serum PSA levels the sensitivity of detection increased [58]. Similarly, Theodorescu et al. identified and verified a signature of 12 peptides using CE-MS analysis of urine from patients with PCa and various benign prostatic conditions. In combination with age, free and total PSA, this signature was found to improve detection [59]. Davalieva et al. performed 2D-DIGE-MS to identify proteins that could distinguish PCa from benign prostate hyperplasia. The authors found a significantly higher abundance of UBE2N and PSMB6 and a significantly lower abundance of PPP1CB in PCa [60]. Another study compared proteomic profiles of fresh frozen tissue sections from patients with PCa and benign epithelial lesions. Using MALDI-MS tissue imaging, the authors identified biliverdin reductase B (BLVRB) to be overexpressed in PCa tissue sections [61]. The emergence of exosomes as a novel source of tumor-specific biomarkers has prompted several independent groups to assess their utility for PCa diagnosis. To determine the utility of exosomal proteins in PCa detection, Duijvesz et al. employed accurate mass and time (AMT) tags coupled to LC-MS/MS profiling in immortalized primary prostate epithelial cells and PCa cell lines. The authors identified PDCD6IP, FASN, XPO1 and ENO1 to be novel candidate biomarkers for PCa detection [62]. A similar study investigated the proteome of urinary exosomes by LC-MS/MS to identify differentially expressed proteins in prostate cancer patients compared to healthy controls. TM256, LAMTOR1, VATL and ADIRF were among 17 up-regulated proteins found to have potential clinical utility for PCa diagnosis [63]. An interesting question to be asked is whether there is a clinical need for diagnostic markers in PCa. In our opinion we are facing a more important problem of patient over diagnosis. Thus, there is a greater need for prognostic rather than diagnostic biomarkers.

Gleason score (GS) is considered the most informative parameter for guiding patient management decisions as it is directly correlated with PCa aggressiveness. However, scoring relies on pathological evaluation of tissue obtained by needle biopsy, which is not always representative and has been a cause of overtreatment [64]. There is an urgent need for molecular markers that can accurately stratify patients. Skvortsov et al. reported differential expression of nineteen proteins in frozen radical prostatectomy tissue from patients with Gleason score 6 and patients with Gleason score 8 to 9. Using MALDI-TOF-MS, the authors identified LAMA1 to be a highly discriminatory marker of low- and high-grade Gleason score tumors [64]. By applying LC-MS/MS, Iglesias-Gato et al. identified prognostic biomarkers able to predict prostate cancer aggressiveness. Pro-NPY was found to be differentially expressed between low and high grade prostate cancer tissue and was associated with an increased risk of PCa mortality, especially in patients with Gleason score ≤ 7 [65]. Another study reported differential expression of proteins in normal prostate epithelial cells, bone metastasis-derived PC-3 cells, and visceral metastasis-derived PC-3M cells by MALDI-TOF-MS. SETDB1 protein was found to be closely associated with PCa prognosis [66]. Moreover, proteomic analysis of formalin-fixed paraffin embedded PCa specimens revealed that ANXA2 expression is predictive of metastatic potential [67]. By applying SELDI-TOF-MS, Al-Ruwaili et al. identified a signature of 26 serum proteins that could accurately distinguish between indolent and aggressive PCa with 73.3% sensitivity and 60% specificity [68]. Analysis of seminal plasma by CE-MS identified a peptide signature that could differentiate indolent from aggressive PCa. Preliminary analysis identified a panel of 11 peptide markers that were able to distinguish between patients with Gleason score 7 and organ-confined (< pT3a) or advanced (≥pT3a) disease [69].

### Bladder cancer

Bladder cancer is one of the most common malignancies affecting the urinary system. Urine has been heavily mined for bladder cancer detection and assessment of aggressiveness due to its direct contact with bladder epithelia [12]. Applying proteomic and peptidomic techniques for the assessment of bladder cancer was shown to identify novel biomarkers for diagnosis and prognosis, as summarized in Table 4.

Cytology is the standard non-invasive detection method for bladder cancer. However, cytology assessment has low sensitivity and specificity, especially for low-grade tumors. Improvements in early-detection would significantly benefit patient management. Two independent studies report elevated levels of urinary APOA1 and APOA2 in bladder cancer. The consistency between the two studies suggests that APOA1 and APOA2 are promising markers for detection. The first study applied iTRAQ and LC-MS/MS to identify differentially expressed proteins in urine from patients with bladder cancer and controls. The authors report elevated levels of SAA4, ProEGF, and six apolipoproteins (APOA1, APOA2, APOB, APOC2, APOC3, and APOE) in patients with bladder cancer [70]. Similarly, Chen et al. report elevated levels of SERPIND1, PRDX2, APOA1 and APOA2 in bladder cancer patients. This group applied iTRAQ labeling and LC-MS/MS profiling to urine of patients with bladder cancer and non-tumor controls. APOA1 was also confirmed by ELISA to have potential value for diagnosis [12]. The same group verified a set of bladder cancer markers they identified by iTRAQ labeling and LC-MS/MS using targeted MRM-MS profiling. The authors verified a panel of six proteins (AFM, ADIPOQ, C4A, APOA2, CP, and F2) that could discriminate bladder cancer from non-cancerous subjects [71]. A similar study applied 2DE-MS/MS to identify differentially expressed proteins in urothelial bladder cancer and adjacent normal tissues. PGAM1 was significantly up-regulated in urothelial bladder cancer. Further analysis found that increased PGAM1 expression was correlated with severity of histological grade [72]. Using iTRAQ labelling coupled to LC-MS/MS, Chen et al. identified 7 proteins with elevated expression in primary bladder cancer compared to adjacent non-tumorous tissue. TAGLN2 was identified as the most promising non-invasive biomarker for bladder cancer screening [73]. Analysis of urine sediments by high-resolution LC-MS/MS identified a multi-parametric signature that could discriminate patients with urothelial carcinoma from normal controls with 90% sensitivity and 67% specificity [74]. In addition, high-resolution MS identified a signature of five urine proteins with 79.2% sensitivity and 93.9% specificity for non-muscle invasive transitional bladder carcinoma diagnosis. In addition, the signature achieved 86.4% sensitivity and 100% specificity for invasive transitional bladder carcinoma diagnosis [75]. Although protein profiling studies have led to the discovery of promising markers for bladder cancer detection, a majority of these studies remain to be validated. Validation is required to determine whether these biomarkers represent a significant addition to current pathological assessment of bladder cancer tumors.

Depending on the depth of invasion, between 25-80% of muscle invasive tumors will progress to metastatic disease [76]. The ability to differentiate indolent and aggressive bladder cancer will significantly improve patient management. To identify prognostic markers of disease, Bansal et al. compared the proteome profiles of serum from patients with low- and high-grade cancer. Using MALDI-TOF-MS, the authors identified two markers, S100A8 and S100A4 that were able to precisely discriminate low- and high-grade cases [77]. Chiang et al. performed LC-MS/MS to characterize the urinary proteome of patients with urothelial carcinoma. SH3BGRL3 was found to be positively associated with higher histologic grading and muscle invasiveness [78]. A large number of urinary peptide markers identified for assessment of aggressive disease appear to be fragments of abundant proteins. The presence of highly abundant proteins strongly influences the urinary peptidome and should be considered in the study design. Bryan et al. demonstrated that alterations in the urine peptidome from patients with and without muscle-invasive urothelial carcinoma can serve as an indicator of progression. The authors identified a panel of 8 peptides derived from abundant serum proteins associated with hematuria (ALB, FGA, FGB, HBA1, and TTR) by MALDI-TOF-MS [79]. Another study aimed at identifying markers of progression compared peptidomic profiles in urine from patients with muscle invasive and non-invasive bladder cancer. Using CE-MS, this group identified a signature of 4 peptides derived from UMOD, COL1A1, COL3A1, and PGRMC1 [80].

## INTEGRATED GENOMICS AND PROTEOGENOMICS

Integrative genomics is an approach that merges different classes of molecular changes, including genomics, epigenetics, mRNA, miRNA, proteomic and peptidomic data to enhance our understanding of cancer and link molecular alterations to cancer phenotypes.

Understanding the interplay between different levels of molecular alterations will open up a new dimension in our understanding of cancer biology and is a cornerstone to better targeted therapy. Using an integrative analysis approach, The Cancer Genome Atlas (TCGA) group analysis suggested that mutations involving the SWI/SNF chromatin remodelling complex could have far-reaching effects on other pathways in RCC [81].

The integration of proteomic and genomic data can give an insight about the cross-talk between proteins. Protein-DNA interactions can identify transcription factors that control gene promoters. Also, comparing mRNA to proteomic data can give insights on splice variations. It can also highlight the involvement of post-transcriptional control of gene expression (e.g. by methylation and miRNAs). Recent evidence have shown that miRNAs and epigenetic control of gene expression go beyond being a one-on-one interaction, but rather to a network that controls an entire biological process with synergetic and mutually exclusive events [82]. Butz et al. employed an integrative genomic/ proteomic approach to identify aryl-hydrocarbon receptor (AHR), grainyhead-like-2 (GRHL2), and KIAA0101 as new pathogenic factors in ccRCC [83]. Girgis et al. applied an integrative genomic approach to identify candidate biomarkers in ccRCC. By combining copy number data from TCGA to previously generated MS protein expression data, the authors were able to identify 12 proteins whose expression significantly correlated with copy number alterations in RCC [84].

Proteogenomics is an emerging field that attempts to facilitate the integration of RNA-seq data and proteomic data derived from tandem MS. The convergence of data through proteogenomics has led to improved genome annotation, identification of genomic aberrations such as single mutations and splice variants, and allowed assessment of the consequence of genomic aberrations on protein expression and (PTMs). Furthermore, proteogenomics allows for the identification of tumor-associated splice variants [85].

## THE JOURNEY TO CLINICAL IMPLEMENTATION

**Figure 3:**
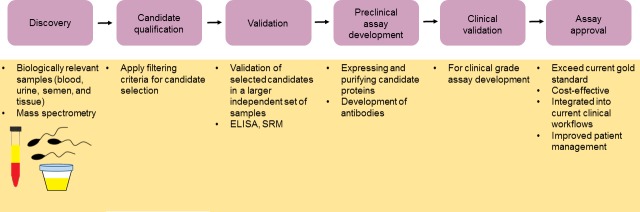
The journey to clinical implementation The introduction of novel cancer biomarkers into the clinic involves an initial discovery phase in which a healthy population is compared to a cancer patient population. Here biologically relevant samples are used as well as appropriate protein array or mass spectrometry technologies. Candidate selection is then achieved using appropriate filtering criteria followed by validation of candidate biomarkers using a large independent set of samples with targeted approaches such as SRM and ELISA. Pre-clinical assay development is followed by clinical validation. Final approval of the assay is obtained provided that the assay exceeds the current gold standard, is cost-effective, can easily be integrated into current clinical workflows, and improves patient management.

The journey of proteomics to clinical implementation involves the discovery of disease-specific proteins in biologically relevant samples, candidate qualification, validation, pre-clinical assay development, clinical validation, and assay approval by health authorities, as shown in Figure 3. In the first phase, the identification of disease-specific proteins is achieved by DNA, protein arrays, or MS. The next phase is biomarker qualification, which involves the use of pre-determined filtering criteria to select a manageable number of candidates. Following qualification, validation in a large number of independent samples is needed to ensure the target molecule is a true biomarker of disease. Validation is commonly achieved using targeted approaches, such as SRM and ELISA. The next step is to develop a pre-clinical assay. This involves expressing and purifying the candidate protein, developing a specific antibody, and validating the final immunoassay in a blinded analysis. The success of a pre-clinical assay then determines whether a candidate biomarker is developed into a clinical-grade assay for subsequent approval [86].

For a new diagnostic test to be implemented into clinical practice several obstacles need to be overcome. Controlled clinical trials should be used to confirm that a novel biomarker meets or exceeds the current gold standards. In addition, the cost-effectiveness of a new assay should be evaluated as well as the strategy to integrate the assay into current clinical work flows. The assessment of whether the novel assay actually improves patient outcomes should also be considered.

Although there has been a push to discover clinically useful protein-based cancer biomarkers, there still remains an unmet clinical need. This may be due to the fact that most newly discovered biomarkers are false discoveries. This is mainly due to the lack of well-designed biomarker studies as well as pre-analytical, analytical, and post-analytical issues which contribute to biomarker failure [87]. Pre-analytical issues include sample collection bias. Analytical issues include false positive identifications, cross-reactivity, and incomplete trypsin digestion. Post-analytical issues include incorrect statistical analysis of data, multiple hypothesis testing, and data over-fitting. Standardization of techniques promises to minimize issues in pre-analytical, analytical, and post-analytical analyses as this would ensure that observed changes in protein abundance are attributable to clinical outcomes and not due to workflow variability [48].

## CONCLUSIONS

It is clear that proteomic and peptidomic strategies represent a promising approach to biomarker discovery. Reports support the potential utility of proteomic and peptidomic techniques for detection of clinically relevant markers in urological malignancies, to help improve patient assessment through early cancer detection, prognosis and prediction of treatment response. The application of proteomic and peptidomic platforms to the assessment of various biological fluids including urine, blood, cerebrospinal fluid and saliva also provides a non-invasive strategy for biomarker identification without the need for tissue biopsy. The ability to validate these markers, through the use of standardized workflows and multicentre participation will be vital for the successful implementation of these markers into clinical practice. The use of next generation MS instruments that display enhanced sensitivity will also play a key role in the success of these platforms. Moreover, the clinical availability of multimarker tests clearly demonstrates that we are moving into an era of clinical proteomics where we rely on a combination of biomarkers rather than a single biomarker. Thus, it may worth focusing on the validation of a combinations of biomarkers which would most likely have clinical utility.

## ABBREVIATIONS

LMW: Low molecular weight
MS: mass spectrometry
RCC: renal cell carcinoma
SRM: selected reaction monitoring
PTMs: post-translational modifications
